# ^18^F-FDG PET-CT Findings Before and After Laparoscopic Cryoablation of Small Renal Mass: An Initial Report

**DOI:** 10.15586/jkcvhl.2015.42

**Published:** 2015-12-10

**Authors:** Brunolf W. Lagerveld, Ferida Sivro, Johan A. van der Zee, Phillippe C. Baars

**Affiliations:** 1 Department of Urology, Onze Lieve Vrouwe Gasthuis, Amsterdam, The Netherlands; 2 Department of Nuclear Medicine, Onze Lieve Vrouwe Gasthuis, Amsterdam, The Netherlands.

## Abstract

The aim of this study was to describe the characteristics of positron emission tomography (PET) molecular imaging combined with low-dose computed tomography (CT) in small renal mass (SRM) treated with cryoablation (CA). Currently, treatment success is defined by the absence of contrast enhancement at CT. However, the use of contrast is relatively contraindicated in patients with renal function impairment, mandating alternative follow-up strategies. Several reasons were identified as criteria for performing PET-CT before and/or after SRM-CA in 9 patients, and the results were retrospectively studied. The histology revealed renal cell carcinoma in 7 patients and oncocytoma in 2 patients. In 6 patients, a PET-CT was performed before and after CA. In one patient, the PET-CT was performed only before CA and in 2 patients only after CA. Before CA, clearly there was metabolic uptake of fluorine-18 fluorodeoxyglucose (^18^F-FDG) in the SRM in all patients. Following CA, the absence of ^18^F-FDG uptakes in the SRM could clearly be noticed. However, the tracer cannot always be distinguished from focal recurrence or reactive inflammatory tissue. In one patient, asymptomatic metastatic bone lesions were noticed when performing PET-CT at follow-up. This pilot study with ^18^F-FDG PET-CT for the follow-up of SRM cryosurgery showed that ^18^F-FDG PET-CT imaging could be used to characterize cryoablative tissue injury at different times after CA.

## Introduction

The number of new renal cancer cases in the Netherlands was estimated to be 2000 in the year 2007 and is expected to increase to 2300 new cases by the year 2020. This increase may be due in part to an increase in the discovery of small incidental solid renal masses (RMs) using cross-sectional imaging (1). Recommended by principal guidelines, nephron-sparing procedures for the management of small RMs have become the standard. Recently, cryoablation (CA) has been added as a viable treatment option for patients with small RM and for those who are at high surgical risk (2–4). Patients who can particularly benefit from thermal ablation procedures are those who are poor surgical candidates because of compromised renal function and/or comorbid disease. However, patients with impaired renal function who are candidates for ablation can be poor subjects for the typical investigational methods used at follow-up.

According to principal guidelines, in practice, there is no consensus on which the set of diagnostic tools, time frame, and frequency of follow-up of the RM-CA are recommended (2, 3). Vascular damage and consequently ischemic injury is one of the mechanisms of action in CA (5). The mainstay of follow-up is the assessment of perfusion in and around the ablated area. Therefore, the current recommendation for follow-up of RM-CA is based on imaging of blood flow. The imaging method selected should be able to evaluate the presence or absence of vital tissue in the ablated area and measure the size of the lesion. Routinely, contrast-enhanced computed tomography (CT) or magnetic resonance imaging (MRI) is used (6). Intravenously administered contrast agents are used to identify contrast enhancement in the target lesion. The observation that the standard follow-up of cryoablated RM in patients with declined renal function is not without the risk of jeopardizing the remaining renal function led to the study of alternative methods of follow-up. The development of contrast-induced nephropathy is a significant complication of intravascular contrast-medium use that is related to excess morbidity and mortality (7, 8). The most important risk factor is preexisting renal impairment that increases the risk of contrast-induced nephropathy by more than 20 times (9). Therefore, alternative imaging, not requiring radiographic contrast medium, should be considered if the alternative imaging adequately addresses the diagnostic questions.

Alternative methods of imaging can focus either on the vascular or on the molecular changes in the CA zone. A method for studying the molecular status of RMs is the positron emission tomography (PET) in combination with low-dose CT, which assesses both RM anatomy and metabolic activity. Fluorine-18 fluorodeoxyglucose (^18^F-FDG) is the most frequently used radiopharmaceutical tracer in PET-CT imaging. However, it is not very accurate in distinguishing renal cell carcinoma (RCC) from the benign solid renal neoplasm. Relatively differentiated cancers such as RCC show faint or no ^18^F-FDG uptake that consequently results in negative PET-CT scans. As a result, ^18^F-FDG is not routinely used in the initial diagnostic workup of solid RM (10). There are nuclear tracers that better distinguish for RCC, but the availability is limited by their short half-life (11, 12). However, ^18^F-FDG PET-CT can possibly be a reasonable alternative to the routine mode of imaging in those patients with relative or imperative contraindications for the use of contrast agents in CT or MRI. This factor makes ^18^F-FDG PET-CT of interest for the follow-up of CA tumors. The hypothesis of this study was that if ^18^F-FDG metabolism could be detected in RM before CA, it should not be detectable in the target zone after CA. The purpose of this study was to describe the spectrum of pre- and postablation ^18^F-FDG PET-CT findings and define their value during follow-up for RM treated with cryosurgery.

## Materials and methods

A total of 9 patients treated with cryosurgery between July 2007 and January 2012 were investigated. All patients were identified with an RM suspected for malignancy. According to the guidelines, patients were informed about optional treatment techniques and each typical follow-up method. All patients consented for CA. The same surgeon performed all laparoscopic CA (LCA) or percutaneously CT-guided procedures. For CA, multiple 17-gauge cryoprobes (Galil Medical, Yokneam, Israel) were used. Histological biopsies were obtained before CA or intraoperatively.

Several reasons were identified as inclusion criteria to prefer PET-CT instead of CT or MRI: renal function impairment, contrast allergy, contraindication for the use of intravenous contrast medium, claustrophobia, metal implants, PET-CT already performed in a referring center, staging of renal cancer, and the identification of a viable tissue metabolism in the case of suspected local recurrence of renal cancer after initial ablative treatment.

Those patients who underwent ^18^F-FDG PET-CT before and/or after RM-CA were retrospectively studied. Patients were identified in our institutional database. The local internal review board approved this study.

### PET-CT technique

^18^F-FDG is a glucose analogue with a positron-emitting ^18^F radioactive isotope substituted for the hydroxyl group at the second position in the glucose molecule. PET imaging of tumors with the FDG tracer is based on the observation that the tumor cells have an enhanced rate of glycolysis compared to the most normal tissue. The uptake of glucose and analogue FDG into malignant cells is facilitated by the increased expression of glucose transporter molecules at the tumor cell surface (13). After intracellular transport, FDG is phosphorylated by hexokinase to FDG-6-phosphate, does not proceed further in the metabolic pathway, and remains trapped in cells. PET identifies this selective focal accumulation of positron-emitting FDG in malignant tumors. The glucose metabolic status can be analyzed quantitatively as the differential uptake ratio and distribution absorption ratio known as the standardized uptake value (SUV) index.

Patients with renal function impairment (GFR-MDRD <60 ml/min/1.73 m^2^) before LCA treatment were offered ^18^F-FDG PET-CT imaging. In case the target lesion showed metabolic activity before CA, the PET-CT was repeated after LCA to assess therapy success. It was postulated that shortly following ablation zone metabolic activity was to be expected at ^18^F-FDG PET-CT within and around the ablation. This would make it difficult to discriminate between inflammatory/reactive tissue and viable tumor tissue. Therefore, the first post-CA PET-CT imaging was performed with a minimum of 3 months following surgery.

According to the local protocol, the treatment success of CA is determined the first time postoperative contrast-enhanced CT imaging is performed and is defined by the absence of (focal) enhancement within the ablated tumor area (14). However, under the circumstances of case-specific indications and awareness of preoperative PET-CT findings, a postoperative contrast-enhanced CT or MRI was not performed in all cases. In several cases, the PET-CT was combined with abdominal ultrasonography. Blood glucose level was measured prior to administering FDG. The plasma glucose level was used to correct SUV measurements. A bolus of ^18^F-FDG (range 141–233 MBq) was intravenously administered 60–90 minutes prior to imaging. A PET scan was performed from the skull base to the groin and combined with a low-dose CT for attenuation correction and anatomic correlation. At preoperative PET-CT imaging, the metabolic uptake was measured in the center of the tumor and the liver for reference. At postoperative PET-CT, the metabolic uptake was calculated in the tumor center, the peripheral rim of the tumor, suspect lesions, and the liver for reference.

## Results

### General results

A total of 9 patients treated with cryosurgery, between July 2007 and January 2012, were retrospectively studied in case preoperative workup and/or follow-up consisted of an ^18^F-FDG PET-CT. Patient characteristics and general surgical information of this study are detailed in **Table 1**. Of the 9 primary tumors that were treated with CA, 7 were diagnosed histologically as malignant and 2 as benign.

**Table 1.** Patient characteristics and demographics

Study number	Age at time of CA (years)	Gender	Tumor size (mm)	Histology	GFR-MDRD	Solitary kidney
1	76	M	45	Clear cell RCC	31	No
2	80	M	44	Clear cell RCC	32	Yes
3	72	M	19	Clear cell RCC	55	Yes
4	68	M	36	Clear cell RCC	61	No
5	64	F	30	Chromophobe RCC	52	Yes
6	79	M	40	Clear cell RCC	65	No
7	64	M	43	Clear cell RCC	33	No
8	70	M	38	Oncocytoma	99	No
9	75	M	48	Oncocytoma	21	No

CA=cryoablation; RCC=renal cell carcinoma; GFR-MDRD=glomerular filtration rate-modification of diet in renal disease.

Indications for PET-CT before and/or after LCA were renal function impairment (n=6), suspicion for local recurrence following LCA (n=1), and contraindication for intravenous contrast-medium use (n=1), and in one patient, a preoperative PET-CT was performed at the referring center for staging purposes.

Before CA, there was clearly metabolic uptake of ^18^F-FDG in the renal tumor in all patients (n=8). In this series, we performed a PET-CT before and after ablative surgery in 6 cases (**Table 2**). It was noticed that the metabolic uptake of ^18^F-FDG can be detected in solid renal lesions, and therefore, this could be used as a reference to discriminate for metabolic activity after RM-CA. In all those cases, we found a significant decrease of metabolic FDG uptake in the center of the cryoablated tumors. However, in the surrounding rim of the ablation area in some patients, clear absence of the ^18^F-FDG tracer could not always be distinguished from focal recurrence or reactive inflammatory tissue.

**Table 2.** In 6 patients, ^18^F-FDG PET-CT imaging was performed before and after cryoablation (CA)

Patient number	SUV renal mass center	SUV renal mass periphery	SUV liver
2a	1.8	1.7	2.6
2b	0.8	3.1	2.4
2c	0.7	3.1	2.6
3a	3.2	2.8	2.8
3b	3.2	2.6	3.2
4a	3.9	3.3	3.2
4b	1.5	2.2	3.0
4c	0.6	0.9	2.3
4d	0.6	0.9	2.7
4e	0.7	0.7	3.2
5a	1.95	1.95	2.95
5b	1.4	2.9	3.2
5c	1.4	2.1	3.0
7a	2.5	2.6	2.8
7b	1.5	2.7	3.2
7c	1.5	2.9	3.4
9a	3.1	3.5	3.7
9b	1.0–1.5	4.5–4.7	3.0

SUV scores are detailed for before CA (a), at first time follow-up (b), and consecutive (c, d, e) follow-up.

### Case reports

Because of the decline in renal function in patient number 1, PET-CT was offered as the method of imaging at initial follow-up. There was no baseline preoperative PET-CT scan performed for comparison. The follow-up PET-CT at 10 months showed no metabolic activity in the center of the ablation zone. However, there was some uptake of FDG around the border of the initial tumor. This could be due to reactive inflammation or rest of tumor activity. The test was not conclusive in view of the absence of a baseline PET-CT. The patient did have consecutive PET-CT imaging at 2 and 3 years follow-up. Both scans reported no evidence of tumor activity in the ablation zone. The activity as noticed at the first post-CA PET-CT was no longer visible. The low-dose CT scans showed a decrease in the size of the ablated tumor. In this case, there was no contrast-enhanced CT or MR scan performed as a control study. At 67 months follow-up, no recurrence or new RM was found using ultrasonography.

**Figure 1.**
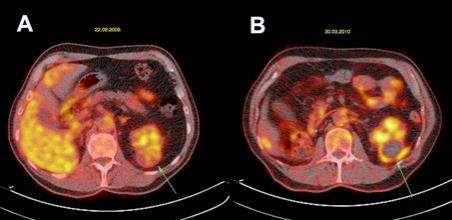
Patient number 2, an example of ^18^F-FDG-distribution in a renal mass before (**A**) and after 5 months cryoablation (**B**) at PET-CT. The light active hypointense uptake in the tumor in the left kidney before cryoablation (green arrow **panel A**) has changed to a hypometabolic area after ablation (green arrow **panel B**). The activity around the ablated tumor in the perirenal fat is possibly due to inflammatory tissue reaction. A contrast-enhanced CT scan as a control showed no enhancement of the ablation zone after treatment.

In patient number 2, the renal function impairment was the reason for a PET-CT scan prior to ablative surgery. Medical history revealed a right radical nephrectomy for RCC. Hypointense (compared to the liver) FDG uptake was noticed in the RM center (**Figure 1A**). Contrast-enhanced CT at 1 month after CA showed a complete ablation of the tumor with some inflammatory reactive tissue in the perirenal fat. Five months post-CA, the PET-CT showed no metabolic activity in the ablation zone. However, some activity was noticed in the perirenal fat covering the outer border of the ablated tumor (SUV 3.1). This activity could be due to reaction or inflammation (**Figure 1B**). A PET-CT scan, performed at 13 months follow-up, again showed no metabolic activity in the ablation zone and again a slight FDG uptake was noticed in the perirenal fat covering the outer border of the ablated tumor. At 40 months follow-up, there was no sign of recurrence as reported by the referring urological center.

The medical history of patient number 3 revealed a radical nephrectomy for RCC and transurethral resection of single metachronous metastasis in the bladder. Follow-up contrast-enhanced CT scan showed an enhancing RM in the solitary kidney. The pre-CA PET-CT showed metabolic FDG uptake in the lesion. During the follow-up PET-CT, 3 months after LCA, there still was an irregular FDG uptake noticed in the ablated tumor. However, the contrast-enhanced CT at 6 months after LCA showed a completely ablated tumor without contrast enhancement. No biopsy was performed to assess recurrence. At 1 year follow-up, there was no evidence of vital RCC at contrast-enhanced CT. Shortly thereafter, the patient was diagnosed with castration-resistant metastatic prostate cancer resulting in his death.

**Figure 2.**
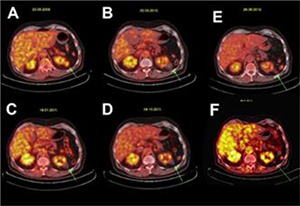
Consecutive ^18^F-FDG PET-CT imaging of a renal cancer before and after cryoablation: Green arrows indicate the tumor or the ablation site in the left kidney of patient number 4. Before surgery, (**A**) there was metabolic activity noticed in the tumor. The low-dose CT scans showed consecutive decrease in the tumor volume, and no metabolic activity was measured in the tumor using ^18^F-FDG PET at 5 months (**B**), 15 months (**C**), and 24 months (**D**) follow-up after LCA. However, at 34 months, newly developed metabolic activity was noticed in the perirenal fat that covered the ablated tumor (green arrow **panel E**). Spontaneous disappearance of metabolic activity in the perirenal fat at 63 months (**panel F**).

Because there was suspicion for macroglobulinemia in patient number 4, there was a contraindication for intravenous contrast-medium usage. The pre-CA PET-CT showed FDG uptake in the RM (**Figure 2A**). After LCA, the suspicion of macroglobulinemia was not proven and a contrast-enhanced CT was performed, which showed complete ablation of the tumor. Five months after LCA, the PET-CT revealed no FDG uptake in the ablated tumor as shown in **Figure 2B**. Also at 15 and 24 months follow-up, the PET-CT scans showed no sign of recurrence (**Figure 2C and D**). However, at 34 months follow-up, the PET-CT revealed FDG uptake at the dorsolateral side of the left kidney in the perirenal fat that covered the ablation area (**Figure 2E**). This uptake was considered suspicious for RCC recurrence, and contrast-enhanced CT confirmed the diagnosis. However, the patient refused biopsy or retreatment. Contrast-enhanced CT at 40 months follow-up revealed the same enhancing tissue in the perirenal fat. However, no other suspicious lesions and no enhancement of the ablated zone were found. The suspected lesion did not increase in size. At repeat contrast-enhanced CT at 47 months follow-up, this lesion had almost disappeared. The follow-up PET-CT scans at 54, 63, and 72 months showed a complete disappearance of FDG uptake in the ablated area, as well as in the perirenal fat covering the ablated zone.

**Figure 3.**
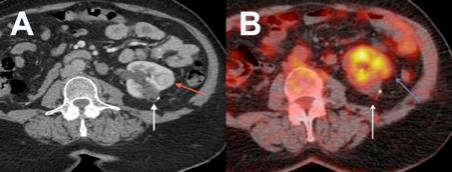
Imaging at 15 months follow-up of patient number 5. **A**, Intravenous contrast-enhanced CT scan in patient number 5 showing a non-enhancing zone (white arrow) at the side of the renal lesion treated with LCA. A second solid lesion with clear contrast enhancement (red arrow) was noticed. **B**, At 18F-FDG PET-CT, the ablation zone showed no metabolic FDG uptake (white arrow). The blue arrow indicates the region of the metachronous RCC with FDG uptake (SUV 3.1).

Patient number 5, with a nephrectomy for RCC in the past, was diagnosed with a suspected malignancy in the solitary kidney. The pre-CA PET-CT revealed a tumor with low FDG uptake. Following CA at 7 months, there was no accumulation of FDG in the ablated tumor. The border of the ablation site showed some metabolic activity that was not suspected to be focal recurrence. The consecutive PET-CT at 16 months follow-up again revealed no metabolic activity in the ablated tumor. Additionally, a renal ultrasound was performed, which showed a decrease in the size of the ablated tumor. However, there was suspicion of a new RM superior from the ablation zone. At control contrast-enhanced CT, there was no enhancement of the ablated tumor. However, near the abated tumor, a new contrast-enhanced RM was noticed (**Figure 3A**), confirming the findings using ultrasound. It was considered a metachronous metastasis and not an evidence for focal recurrence after LCA. This tumor was almost completely endophytic and initially not recognized at follow-up PET-CT. The patient underwent a successful CT-guided percutaneous CA for this second lesion. The histology confirmed RCC. At 15 months following the second CA, 2 new metachronous lesions were diagnosed at contrast-enhanced CT and successfully treated with angio-beam-CT-assisted percutaneous CA.

**Figure 4.**
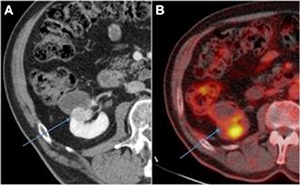
Local RCC recurrence of RM-CA. At 8 months following LCA for patient number 6, a focal contrast-enhanced area at the posterior side within the ablation zone was noticed on contrast-enhanced CT scan (**A**, blue arrow). Although not as sharply distinctive as the contrast-enhanced CT scan, we noticed metabolic activation in the same posterior rim area at 18F-FDG PET-CT scan (**B**, blue arrow).

There was no PET-CT performed prior to LCA in patient number 6. The first follow-up contrast-enhanced CT at 3 weeks after LCA showed complete ablation of the tumor. However, the consecutive scan at 7 months showed new focal enhancement in the dorso-central rim of the initially CA tumor suspected for recurrence (**Figure 4A**). At PET-CT, the center part of the tumor showed no FDG uptake (SUV 1.2–1.5) compared to the liver as reference (SUV 2.8). However, the dorso-central area of the initial ablated tumor showed metabolic activity (SUV 2.7–2.8). This FDG uptake could be related to an inflammatory reaction, focal recurrence, or the accumulation of FDG in the collecting system (**Figure 4B**). The finding of focal enhancement at the contrast-enhanced CT was decisive for offering consecutive treatment. This recurrence was successfully treated with CT-guided percutaneous CA. Histological biopsy revealed recurrence of vital RCC tissue. Follow-up with contrast-enhanced CT up to 43 months after the retreatment still shows no signs of recurrence.

**Figure 5.**
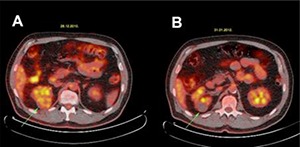
FDG patterns before and after RM-CA in patient number 7. Before treatment (**A**), the renal tumor in the right kidney shows a low FDG uptake at ^18^F-FDG PET-CT. The PET-CT scan after treatment (**B**) shows the same tumor without signs of the existence of vital tissue. Green arrows indicate the tumor before (**A**) and after (**B**) cryoablation.

**Figure 6.**
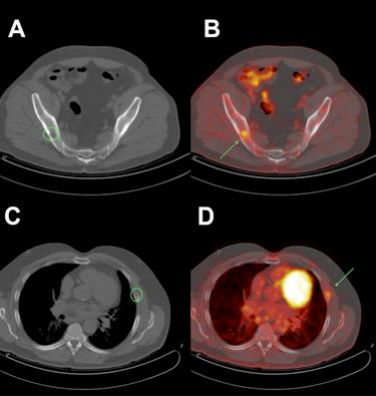
Panel of ^18^F-FDG PET-CT imaging at 6 months follow-up of patient 7. There are 2 FDG-avid bone localizations strongly suspected of being metastases (**A, B**). The low-dose CT scan without contrast shows cystic lesions in the right iliac bone (**A**, green circle) and in the fifth left costal rib (**C**, green circle). Both cystic locations correlated with the “hot spot” FDG uptake as shown in **B** and **D** (green arrows).

Patient number 7 was offered a PET-CT because of poor renal function before treatment of a suspected RM. At the center of this tumor, a low FDG uptake was found at the pre-CA PET-CT (**Figure 5A**). The patient underwent LCA via a retroperitoneal approach. At contrast-enhanced CT, performed 2 weeks following surgery, the tumor showed consistent enhancement of the superior-anterior part that was considered an incomplete ablation with persistent vital tumor as a result. Consequently, the LCA was repeated by intraperitoneal approach. The PET-CT at 6 months post-LCA showed the now 2 times CA RM without clues for vital tumor tissue (**Figure 5B**). However, now there were 2 FDG-avid bone localizations that were strongly suspected to be osteolytic metastases as is shown in **Figure 6**. The contrast-enhanced CT as control and for further dissemination study showed no particular enhancement of the ablated tumor. The bone metastases were treated with external beam radiation. During the follow-up, pulmonary metastases also developed, and palliative systemic treatment was administered. The patient died 2 years later of systemic disease.

Patient number 8 was referred for LCA treatment of an incidentally discovered RM in the left kidney. The referring center performed an ^18^F-FDG PET-CT for dissemination purposes and showed a metabolically active (SUV 5.7) tumor of the left kidney. The patient underwent LCA. The histology obtained by intraoperative biopsies revealed oncocytoma. Because of the benign nature of this RM, no postoperative PET-CT was performed. The postoperative contrast-enhanced CT showed a complete ablation of the RM.

Patient number 9 was offered PET-CT prior to LCA because of poor renal function. In case of proven malignancy, we could offer follow-up using PET-CT. At pre-CA PET-CT, there was FDG uptake in the tumor located in the left kidney. Even though the intraoperative biopsies revealed a histological diagnosis of oncocytoma, the patient was offered a PET-CT to define treatment success. After CA, in comparison with the PET-CT before CA, there was a change in the image of the tumor in the left kidney, with a recognizable pattern of isometabolic to no metabolic activity. There was no contrast-enhanced CT performed as control.

## Discussion

In 2005, the first report of using fused PET-CT imaging for the follow-up of an RM treated with CA was published (15). This case report showed that a decrease of molecular activity was found using PET-CT in the RM after cryosurgery compared to that using PET-CT before surgery. The purpose of this study was to determine whether tracer uptakes found at baseline and no tracer uptake at follow-up in ^18^F-FDG PET-CT could be used to assess the response to CA for RCC. We noticed that solid tumors could be detected as metabolically active lesions at ^18^F-FDG PET-CT imaging. However, the ^18^F-FDG PET-CT is not routinely used in the initial diagnostic workup of solid RM because it does not discriminate between benign and malignant renal growth (10). Hence, this study showed metabolic activity in oncocytoma and RCC. The number of cases was too limited to be able to assess a possible difference in the SUV for benign and malignant RM. However, this study showed that ^18^F-FDG uptake can be detected in solid renal lesions. Therefore, this could be used as a reference to discriminate for metabolic activity after RM-CA. In all those cases, we found a significant decrease of metabolic FDG uptake in the center of the CA tumors. Therefore, we postulate that ^18^F-FDG PET-CT imaging can be used as an alternative imaging method to contrast-enhanced CT/MRI for the assessment of treatment success of LCA.

In all cases, there was a relative or imperative reason for the use of an alternative imaging technique other than intravenous contrast-medium imaging to assess the renal tumor viability before and after CA. One of the major benefits of ^18^F-FDG PET-CT is that there are no contraindications for patients with decreased renal function. The patient’s height, body weight, and medical history of diabetes mellitus are essential for the interpretation of SUV measurements (16). FDG-PET and CT are imaging modalities that have been validated in routine clinical practice. Integrated PET/CT combines PET and CT in a single imaging device and allows morphological and functional imaging to be carried out in a single procedure. This integration has advantages for the assessment of the vitality of a small RM before and after CA. Vascular damage and consequently ischemic injury is a significant mechanism of action in CA (5). The current mainstay of follow-up is the assessment of vascular flow in and around the ablated area. Therefore, the recommendations for follow-up of CA for a small renal tumor are based on imaging of blood flow (14). However, identifying the absence of metabolic activity using PET can also assess the viability of tissue. Another morphological feature is that CA renal tumors tend to decrease in size over time. At 3 years of follow-up of patients treated with LCA, Gill et al. (17) observed a gradual involution of the ablation zone diameter by an average of 75%. Furthermore, 38% of the ablation zones were undetectable on MRI after 3 years follow-up. Even though low-dose non-contrast-enhanced CT is not intended for radiological diagnosis, it makes it possible to see a decrease in the size of most ablated tumors over time.

One of the limitations of this study was the resolution achieved by PET-CT. It does not allow for accurate interpretation of metabolic activity at the rim borders of the ablation zone. The measurement of SUV in an area selected <1 cm diameter becomes inaccurate and, therefore, selection of the region of interest is of utmost importance. However, at a short distance, a focal recurrence can be surrounded by FDG concentrating morphological structures, such as the normal renal parenchyma and/or the urinary collecting system. Artifacts as a result of high FDG concentration in the urine can be minimized by adequate prehydration. In patient number 6, FDG accumulation was found in the rim of the ablation zone at the dorso-central border. Initially, this was not recognized as a suspect for focal recurrence. Contrast-enhanced CT showed focal enhancement and, therefore, the patient underwent consecutive biopsy and cryosurgery. Histology confirmed the presence of viable RCC tissue. The post-LCA PET-CT of patient number 3 revealed irregular FDG accumulations in the ablation zone. However, contrast-enhanced CT 3 months later showed no evidence of enhancement in the ablated tumor. In this case, the limited tumor dimension (19 mm diameter) possibly hampered the interpretation of the PET-CT findings.

After LCA, in 4 more patients, metabolic activity was noticed at the rim of the ablation assessed by ^18^F-FDG PET-CT. In patient number 4, this was found in the peritumoral fat by the third consecutive PET-CT almost 3 years post-LCA, whereas previous scans showed no peripheral metabolic activity. Therefore, this must be considered a suspect for metastases. However, this is not confirmed by histology. In patient numbers 1, 2, and 5, the metabolic activity of the peripheral rim was encircling the metabolic inactive ablation zone as noticed in the first PET-CT following CA. This seems comparable to the literature where findings by CT and MRI of rim enhancement were noticed around the ablation zone in 17–30% of cases at 1 month after treatment (18–20). This periablational enhancement is considered benign. It is suggested that this is a physiologic response to thermal injury, and it appears as a relatively concentric, symmetric, and uniform process with smooth inner margins (6). It seems that during the first month after ablation, this finding is of no consequence; however, if it were to occur later at follow-up, it could be interpreted as a sign of recurrence and will typically appear as irregular peripheral enhancement. The PET-CT findings of patient number 4 can possibly be interpreted as such.

In this series, the PET-CT cross-sectional area stretched between the skull base and the groin. Therefore, using this technique, more than the local ablation site could be studied. The cross-sectional area of the thorax and abdomen, in combination with integrated method of imaging, enables us to locate metabolically active pulmonary, lymph node, or bone metastases. In patient number 7, 2 asymptomatic bone metastases were found this way. However, these lesions could also have been noticed during a cross-sectional contrast-enhanced CT of this area.

The search for the best method to assess treatment success after cryosurgery for RMs remains subject to new investigations. However, in patients with relative or imperative contraindications for the use of contrast agents in CT or MRI, ^18^F-FDG PET-CT may be a reasonable alternative in the follow-up in CA of RMs. The total accumulated radiation dose of consecutive CT scans performed is considered negatively related to the follow-up of LCA. The radiation dose with PET-CT is lower compared to the radiation dose of a full diagnostic cross-sectional CT scan. The radiation dose of 185 MBq ^18^F-FDG is about 3–4 mSv.

To our knowledge, this pilot study is the first to assess ^18^F-FDG PET-CT imaging, reviewing the spectrum of pre- and post-ablation PET-CT findings, and to discuss its value in the follow-up of renal tumors treated with cryosurgery. However, this study has, as have most pilot studies, many limitations. Only a few patients, at different times after CA, were studied to examine the possibility of using ^18^F-FDG PET-CT imaging to characterize the lesion. No longitudinal data were collected, and no direct comparison with CT/MRI was intended. Furthermore, the majority of PET-CT findings are not confirmed by histology and, therefore, the accuracy could not be assessed. However, the results of this study show that ^18^F-FDG PET-CT can be used in the follow-up in patients with small RM that underwent CA. We assume that this justifies a larger prospective and comparative study to determine the exact value of this technique for assessing treatment success and detecting recurrent tumors after CA.

## Conclusion

This experience with the ^18^F-FDG PET-CT for the follow-up of RM-CA showed that ^18^F-FDG PET-CT imaging could be used to characterize CA tissue injury at different times after CA. A longitudinal prospective study, comparing ^18^F-FDG PET-CT imaging to CT/MRI and confirmed by histology, is needed to establish its exact value in the follow-up of RM-CA.

## References

[1] Pantuck AJ, Zisman A, Belldegrun AS. (2001). The changing natural history of renal cell carcinoma.. J Urol.

[2] Campbell SC, Novick AC, Belldegrun A, Blute ML, Chow GK, Derweesh IH (2009). Guideline for management of the clinical T1 renal mass.. J Urol.

[3] Ljungberg B, Cowan NC, Hanbury DC, Hora M, Kuczyk MA, Merseburger AS (2010). EAU guidelines on renal cell carcinoma: the 2010 update.. Eur Urol.

[4] Klatte T, Mauermann J, Heinz-Peer G, Waldert M, Weibl P, Klingler HC (2011). Perioperative, oncologic, and functional outcomes of laparoscopic renal cryoablation and open partial nephrectomy: a matched pair analysis.. J Endourol.

[5] Hoffmann NE, Bischof JC. (2002). The cryobiology of cryosurgical injury.. Urology.

[6] Kawamoto S, Solomon SB, Bluemke DA, Fishman EK. (2009). Computed tomography and magnetic resonance imaging appearance of renal neoplasms after radiofrequency ablation and cryoablation.. Semin Ultrasound CT MR.

[7] Levy EM, Viscoli CM, Horwitz RI. (1996). The effect of acute renal failure on mortality. A cohort analysis.. JAMA.

[8] Rihal CS, Textor SC, Grill DE, Berger PB, Ting HH, Best PJ (2002). Incidence and prognostic importance of acute renal failure after percutaneous coronary intervention.. Circulation.

[9] Rudnick MR, Goldfarb S, Wexler L, Ludbrook PA, Murphy MJ, Halpern EF (1995). Nephrotoxicity of ionic and nonionic contrast media in 1196 patients: a randomized trial. The Iohexol Cooperative Study.. Kidney Int.

[10] Aide N, Cappele O, Bottet P, Bensadoun H, Regeasse A, Comoz F (2003). Efficiency of [(18)F] FDG PET in characterising renal cancer and detecting distant metastases: a comparison with CT.. Eur J Nucl Med Mol Imaging.

[11] Perini R, Pryma D, Divgi C. (2008). Molecular imaging of renal cell carcinoma.. Urol Clin North Am.

[12] Divgi CR, Pandit-Taskar N, Jungbluth AA, Reuter VE, Gönen M, Ruan S (2007). Preoperative characterisation of clear-cell renal carcinoma using iodine-124-labelled antibody chimeric G250 (124I-cG250) and PET in patients with renal masses: a phase I trial.. Lancet Oncol.

[13] Bensinger SJ, Christofk HR. (2012). New aspects of the Warburg effect in cancer cell biology.. Semin Cell Dev Biol.

[14] Goldberg SN, Grassi CJ, Cardella JF, Charboneau JW, Dodd GD, Dupuy DE (2005). Image-guided tumor ablation: standardization of terminology and reporting criteria.. Radiology.

[15] Wagner AA, Solomon SB, Kavoussi LR. (2005). Imaging following cryoablation of a renal lesion.. Nat Clin Pract Urol.

[16] Boellaard R O’Doherty MJ Weber WA Mottaghy FM Lonsdale MN Stroobants SG FDG PET and PET/CT: EANM procedure guidelines for tumour PET imaging: version 1.0. Eur J Nucl Med Mol Imaging 2010 37 1 181–200 http://dx.doi.org/10.1007/s00259-009-1297-4 19915839 10.1007/s00259-009-1297-4 PMC2791475

[17] Gill IS, Remer EM, Hasan WA, Strzempkowski B, Spaliviero M, Steinberg AP (2005). Renal cryoablation: outcome at 3 years.. J Urol.

[18] Remer EM, Weinberg EJ, Oto A, O’Malley CM, Gill IS. (2000). MR imaging of the kidneys after laparoscopic cryoablation.. AJR Am J Roentgenol.

[19] Bolte SL, Ankem MK, Moon TD, Hedican SP, Lee FT, Sadowski EA (2006). Magnetic resonance imaging findings after laparoscopic renal cryoablation.. Urology.

[20] Rutherford EE, Cast JE, Breen DJ. (2008). Immediate and long-term CT appearances following radiofrequency ablation of renal tumours.. Clin Radiol.

